# Dieulafoy’s disease of the bronchus: rare but potentially fatal: a case report and a review of literature

**DOI:** 10.1186/s13019-023-02242-0

**Published:** 2023-07-04

**Authors:** Salsabil Daboussi, Marwa kacem, Nouha Boubaker, Mariem Chaabene, Chiraz Aichaouia, Samira Mhamdi, Zied Moatemri

**Affiliations:** The pulmonology department of the military hospital of Tunis, Tunis, Tunisia

**Keywords:** Hemoptysis, Dieulafoy’s disease, Bronchi, Embolization, Surgery

## Abstract

**Background:**

Dieulafoy’s disease of the bronchus can cause massive and even fatal hemoptysis. Even though it is rare, it should be considered by physicians all over the world. This paper reports a case of bronchial Dieulafoy’s disease and summarizes the data of similar cases reported in literature.

**Methods:**

We report a case of bronchial Dieulafoy’s disease (BDD) in Tunisia. We also present a review of literature related to BDD from 1995 to 2022 using the PubMed, Google Scholar, web of science and Chinese National Knowledge Infrastructure Databases. Clinical characteristics, chest imaging, bronchoscopic and angiographic findings were summarized. Treatment courses were identified as well as patients’ outcome.

**Results:**

We report the case of a 41-year-old man, so far in good health, presenting with massive hemoptysis. Bronchoscopy showed blood clots and a protruding lesion covered by mucosa with a white pointed cap at the entrance of the right upper lobe. Biopsies were not attempted. Embolization of bronchial artery was first realized and was not successful, with post procedure complications. Surgical intervention stopped the bleeding and pathological examination of the resected specimen confirmed Dieulafoy’s disease of the bronchus. Ninety cases of BDD were reported from 1995 to 2022. The main symptom was hemoptysis. Chest imaging findings were not specific. The diagnosis of BDD was mainly based on the bronchoscopy, branchial angiography and pathological findings or surgical specimens. Bronchoscopy findings were mostly nodular or prominent lesions (52.4%). Twenty-eight patients underwent bronchoscopic biopsies, 20 had massive bleeding and 10 died. Bronchial angiography mainly showed tortuous and dilation of bronchial artery, and the lesions were mainly located in the right bronchus. Selective bronchial artery embolization (SBAE) was performed in 32 patients and 39 patients underwent surgery.

**Conclusion:**

To our knowledge, this is the first case of bronchial Dieulafoy’s disease to be reported in Tunisia and North Africa. When the diagnosis is suspected, bronchoscopic biopsy should be avoided as it might lead to fatal hemorrhage. Selective bronchial artery embolization can stop the bleeding, but surgery can be required.

## Background

Massive hemoptysis is a medical emergency which is still feared by most physicians. It presents several diagnostic and therapeutic challenges. Determining the origin of bleeding and underlying etiology is a cornerstone of the treatment plan. However, it may not be immediately apparent and a thorough investigation must be lead. We present the case of a young patient suffering from massive hemoptysis due to bronchial Dieulafoy’s disease, as well as a review of literature of similar cases in order to improve the understanding of the disease.

## Case presentation

A 41-year-old man was admitted in February 2022 to the pneumology department with sudden onset of massive hemoptysis.

He had a less severe episode of hemoptysis one year ago, concomitant to dental extraction, but was otherwise healthy. He worked as a university professor and was an occasional smoker. Initial clinical examination was normal, aside from sinus tachycardia. Blood biochemistry parameters showed a decrease in hemoglobin level from 14 g/dl to 9 g/dl, indicating blood transfusion.

**Fig. 1:**
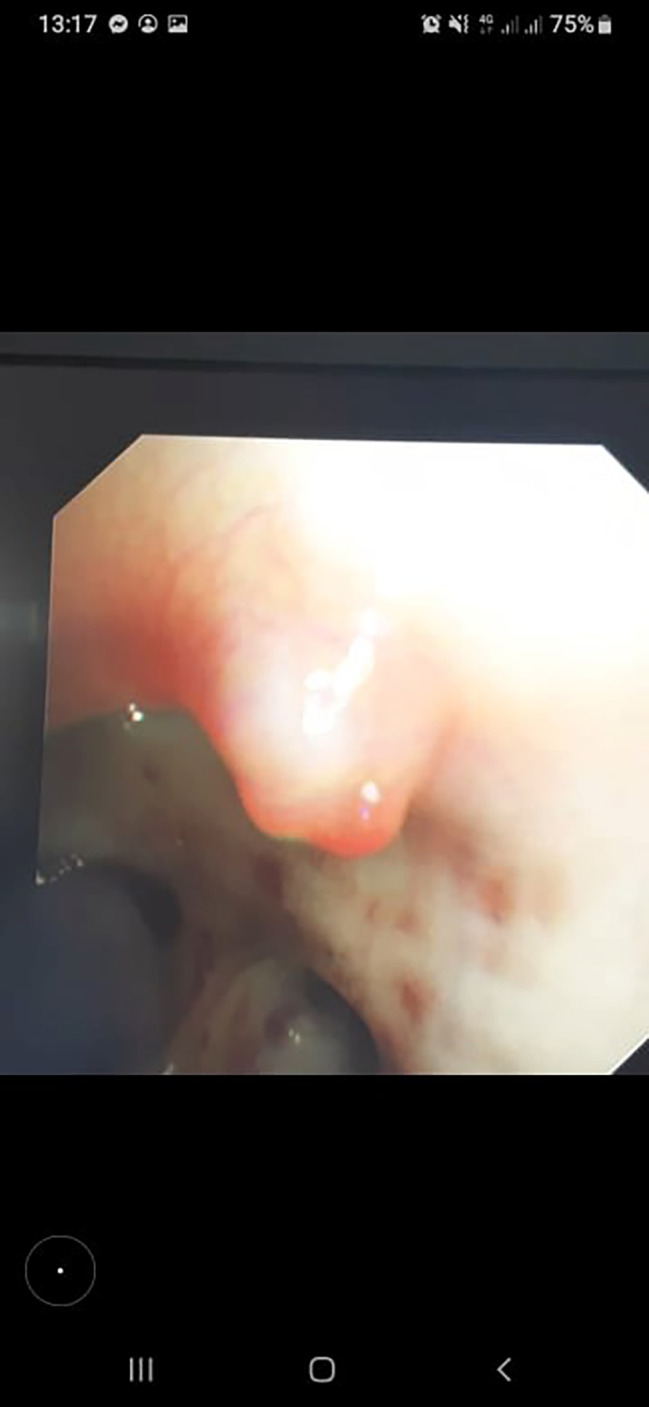
Nodular protrusion from the mucosa at the entrance of the right upper lobe

**Fig. 2:**
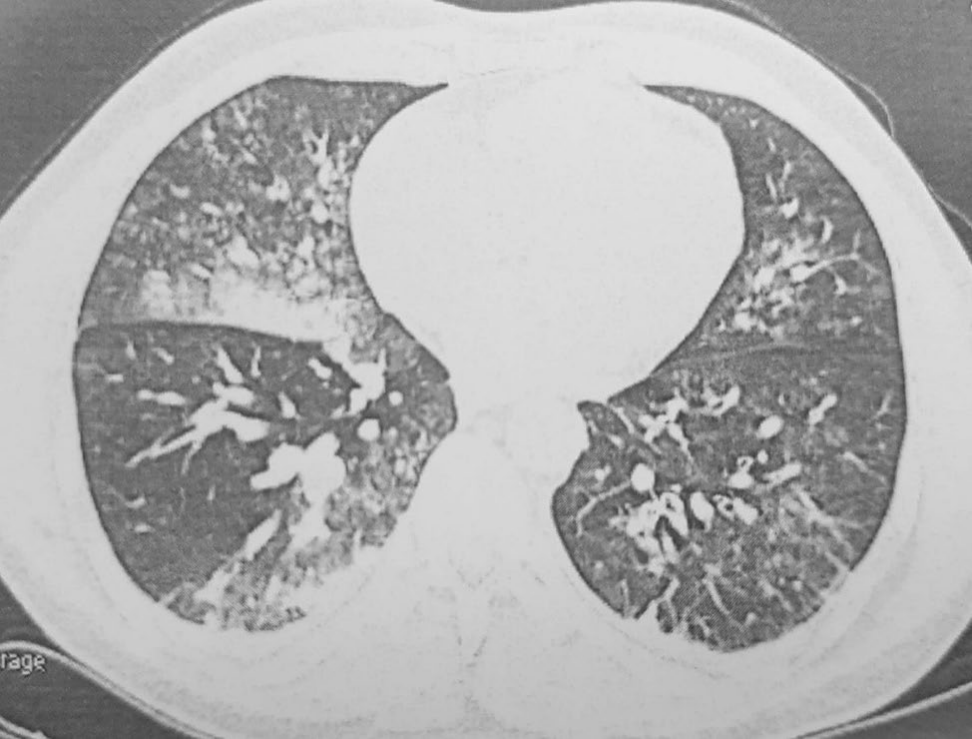
Ground glass opacities of both lungs in computed tomography scans

Bronchoscopy showed bleeding stigma in the right main bronchi and a protruding lesion at the entrance to the right upper lobe. The surface was covered by mucosa and had a white pointed cap (Fig. 1). Blood clots were also noted in the right lower lobe bronchus. Computed tomography (CT) scans revealed ground glass opacities at the upper, middle and lower lobes of the right lung (Fig. 2). The right bronchial artery had an ectopic origin from the aortic arche. Further workup with bronchial arteriography revealed no tortuous arteries nor any vascular blush. However, the patient was still coughing up important amounts of fresh blood, approximately 300 ml in an episode, despite prescribing systemic hemostatic treatment.

All of the investigations performed found no evidence of any etiology and vasculitic screen was negative.

**Fig. 3:**
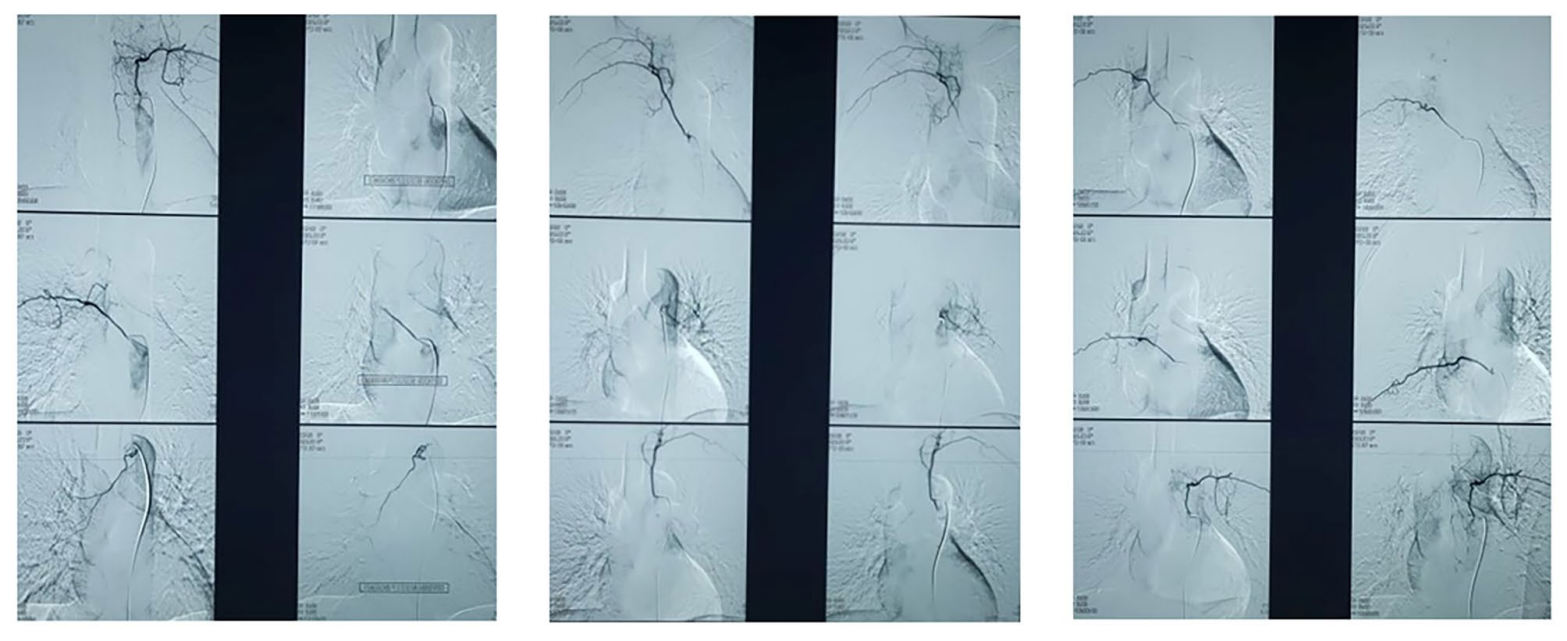
Angiographic arterial embolization

Given the findings of the bronchoscopy, we performed an arterial embolization of the right bronchial artery. The left intercostal artery, the left cervico-thoracic artery and the 5th right intercostal artery gave vascular branches to the right hilum and embolization of these arteries was also performed (Fig. 3). However, the procedure failed to prevent the recurrence of his bleeding. The patient developed complications afterwards. He suffered from splenic infarction, bilateral renal infraction and a posterior inferior cerebellar artery stroke, due to spilling of the embolizing agent. The patient also developed acute respiratory failure due to bilateral pulmonary embolism, proximal on the left side. Ultimately, he required hemostasis surgery.

He had a right pneumonectomy on veno-venous extracorporeal membrane oxygenation with simple surgical follow up. Curative heparin was prescribed to treat his pulmonary embolism.

**Fig. 4:**
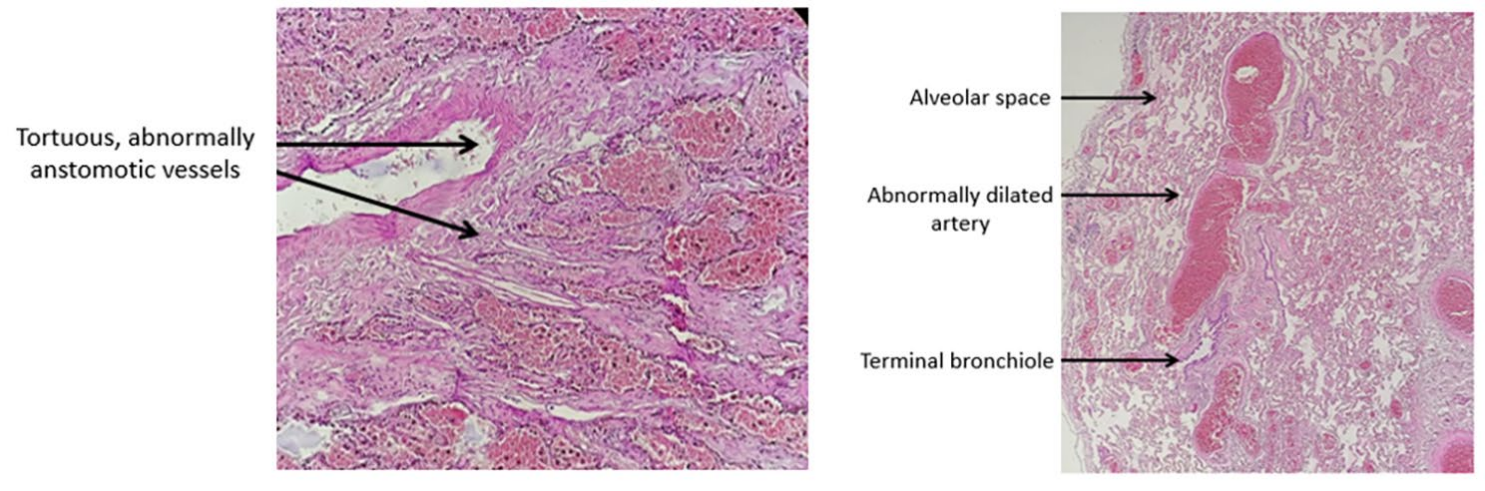
Pathological findings: Abnormally dilated, sinuous and anastomotic vessels extending into the bronchial mucosa

The pathological examination of the resected specimen had found abnormally dilated, sinuous and anastomotic vessels extending into the bronchial mucosa consistent with the diagnosis of bronchial Dieulafoy’s syndrome (Fig. 4).

The postoperative course was uneventful aside from a chest wall hematoma. A follow-up 4 months later, the patient was well with no further episodes of hemoptysis.

## Discussion

Dieulafoy’s disease has been first reported in 1898 by Georges Dieulafoy usually affecting the digestive tract [1]. The bronchial location of this disease (BDD) has been first reported by Sweerts et al. in 1995 [2]. It’s an extremely rare affection which may manifest by massive hemoptysis. Over the past decade, cases of the disease have been increasingly outlined. A recent systematic review of the literature published by Qian et al. collected 73 cases from 1995 to 2019 [3]. We reported a case of BDD and searched the databases of PubMed, Google Scholar, Web of science and Chinese National Knowledge Infrastructure using the terms “bronchial Dieulafoy’s disease or Dieulafoy’s disease of the bronchus”. After removing repeated cases and incomplete ones, we identified 62 articles and 90 cases [2–64] from January 1995 to December 2022. To our knowledge, this case would be the first one reported in Tunisia and North Africa.

**Fig. 5:**
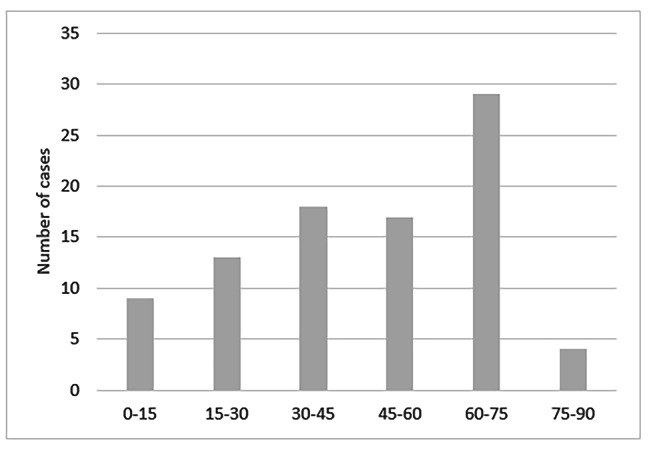
Distribution by age of 90 cases of Dieulafoy’s disease

The cause of the disease is still unknown. Theories vary from congenital vascular malformations to bronchial injury secondary to previous infections. Parrot et al. [15] suggested a possible association with inflammatory lesions in tuberculosis or stretching and dilation of the bronchial artery. Advanced age and tobacco smoking have been implicated in the increase of bleeding-related complications [27]. However, the disorder may affect people at every age especially middle-aged adults [3], and also non-smokers. There were 34 females and 56 males in 90 patients reported in this review with a male to female ratio of 1.6. Their age varied form 9 months to 85 years old (Fig. 5). History of smoking was found in 38 cases and previous respiratory diseases are summarized in Table 1.

**Table 1:** General information of patients (N = 90)

General information	Number of cases	Proportion (%)
Smoking history		
Not mentioned Yes No	21 38 31	23.3 42.2 34.4
Respiratory diseases		
Tuberculosis Chronic obstructive pulmonary disease Repeated infection Bronchiectasis Asthma Pulmonary hypertension Pulmonary fibrosis	12 9 8 6 2 1 1	13.3 10 8.9 6.7 2.2 1.1 1.1
Clinical manifestations		
Hemoptysis Cough Dyspnea or acute respiratory failure Pulmonary infection Fever Chest pain	74 19 11 4 4 1	82.2 21.1 11.1 4.4 4.4 1.1

Clinical manifestations are non-specific, but the most common one was recurrent hemoptysis (Table I). Massive and even fatal hemoptysis may occur especially while performing bronchoscopy guided biopsy. Other symptoms such as a cough [24, 30, 45, 57], chest pain [28], infection [17, 25, 45] or respiratory failure [4, 41, 43, 46] can be reported by patients.

Establishing the diagnosis of Dieulafoy’s disease can be quite difficult. An exhaustive evaluation was lead with chest X-rays, computed tomography (CT) scans, bronchoscopies, biopsies and bronchial angiographies in historical cases.

In Dieulafoy’s disease of the bronchus, chest X-rays and chest CT are rarely contributive to the diagnosis. The relevance of this exam is its contribution to excluding other lung diseases causing the bleeding. In this review, Chest X-ray was performed for 38 patients. Whereas, 79 patients had chest CT. They showed mostly manifestations of an intrapulmonary hemorrhage with ground glass opacities (24 cases) and inflammatory changes (34 cases). Other findings were atelectasis (10 cases), consolidation (5 cases), bronchiectasis (10 cases) and endobronchial nodes or masses (6 cases). Due to lack of specificity and sensitivity, most authors agree that chest X-rays and CT scans are not the best modality to diagnose Dieulafoy’s disease.

Bronchoscopy was performed for most patients (84 cases). It mainly showed a mucosal protrusion in the site of the bleeding bronchus, which diameter can be only a few millimeters [3, 64]. The mucosa covering the protruding surface may look like a “white cap”, without pulsations. The surrounding mucosa can be normal or congested and other lesions can be found (Table 2).

**Table 2:** Bronchoscopy findings (N = 84)

Bronchoscopy findings	Number of cases	Proportion (%)
Endobronchial hemorrhage Blood clots or thrombus Nodular or prominent lesions Only a white cap Non pulsatile process Normal	7 16 44 3 8 8	8.3 19 52.4 3.6 9.5 9.5

These bronchoscopic findings in BDD are not always diagnostic since the abnormal vessel is usually pinpoint mucosal defect surrounded by normal-looking mucosa. Moreover, a small lesion can be undetected due to pooling of blood or clots within bronchial lumen or to a distal localization below the subsegmental bronchus. Bronchial angiography can contribute to the diagnosis as it can show a rich blood supply to the corresponding site of the lesion; a deformed, tortuous and dilated artery with signs of bleeding [23]. Also, when detected, these abnormalities can indicate a selective bronchial embolization, which has an important therapeutic value. However, lesions of the arteries may not be visualized, as it was the case for 2 cases in the literature [3] and for our patient. The bronchial arteries usually originate from the proximal descending thoracic aorta. Arteries that originate elsewhere in the aorta or from other vasculature are termed ectopic [65]. Right bronchial artery occasionally originates from the aorta but more commonly shares its origin with another artery, usually an intercostal artery. Choi et al. [66] evaluated in their study the spectrum of variations in bronchial artery and among ectopic origins, concavity of the aortic arch was the most common.

In most cases, the abnormal lesions were located in the right bronchus (64 cases), which may be due to its anatomical structure. The diversity of embryonic development of the right bronchial artery accounts for a higher risk of abnormalities causing the congenital BDD [5]. Cases of BDD of left bronchus and bilateral bronchi were also reported (Table 3). In most cases, bleeding originated from the bronchial artery system [3].

**Table 3:** Lung segment localization of bronchial Dieulafoy’s disease (N = 90)

Lung segment localization	Number of cases	Proportion (%)
Right bronchus • Right main bronchus • Right upper lobe bronchus • Right intermediate bronchus • Right middle lobe bronchus • Right lower lobe bronchus	64 3 16 7 21 24	71.1 3.3 17.8 7.8 23.3 26.7
Left bronchus • Left main bronchus • Left upper lobe bronchus • Left lower lobe bronchus	25 5 10 10	27.8 5.6 11.1 11.1
Bilateral bronchus	5	5.6

Among 90 cases reported, biopsies were attempted for 28 patients whom presented a nodular lesion without a typical vascular lesion. It was primarily suspected to be an endobronchial mass or carcinoid tumor. Twenty patients had severe hemoptysis [4, 6, 11, 17–19, 21, 24, 25, 30, 36, 37, 45, 48, 49, 52, 57, 61–63] and ten patients did not survive [4, 6, 17, 24, 25, 30, 45, 49, 52, 61].

Bronchial biopsies in such diseases entail the risk of triggering fatal hemoptysis. Since 2014, with a better understanding of Dieulafoy’s disease, biopsies have been avoided for nodules suspected to be caused by Dieulafoy’s disease [26], which has reduced the risks of massive hemorrhage.

In 2010, Guiroli et al. [19] have demonstrated the clinical utility of endobronchial ultrasound EBUS in the evaluation of bronchial alteration suspicious of Dieulafoy’s lesion. This technique can be helpful to clarify the nature of the nodular lesion and contributes to the diagnosis, avoiding potentially disastrous interventions [32]. The major manifestation is a fluid echo-free zone in the submucosal lesion. The Doppler mode can be used to detect blood flow. However, convex probe EBUS cannot reach the upper lobe bronchus nor segmental bronchus. Radial probe EBUS can be used instead but it has no doppler mode and cannot determine blood flow within the lesion.

To make a definite diagnosis, many researchers consider that pathological examination of biopsies, surgical or autopsy specimens is required. However, there are no uniform diagnostic criteria and due to risks involved, the need for pathological diagnosis remains controversial. In some cases, the diagnosis was only based on the findings of bronchoscopy and bronchial angiography [28, 29, 31].

The pathological exam usually shows an arterial malformation in the bronchial submucosa. The tortuous, dilated and deformed artery forms small nodules coated with bronchial mucosa and protruding from the bronchial lumen [10, 15]. Diagnosis is confirmed when a dysplastic artery is identified in the bleeding territory without evidence of other underlying lung disease, vasculitic changes or neoplasm.

Treatment options include medical treatment, surgical lung resection, selective bronchial artery embolization (SBAE) and bronchoscopic ablation. Among 90 patients reported, 32 patients were treated with only SBAE, whereas, 15 patients had pulmonary lobectomy (Table 4). Two patients were successfully treated with argon plasma coagulation through bronchoscope [29, 39]. One patient failed to receive cryotherapy, and then placed silica gel stent [14].

**Table 4:** Treatment of bronchial Dieulafoy’s disease (N = 90)

Treatment plan	Number of cases	Proportion (%)
Medical treatment	9	10
Selective bronchial artery embolization only	32	35.6
Selective bronchial artery embolization (unsuccessful) then surgery (lobectomy)	15	16.7
Selective bronchial artery embolization (successful) then surgery (lobectomy)	9	10
Surgery only	15	16.7
Bronchoscopic treatment		
Freeze + place silica gel stent Argon ion coagulation Endotracheal intubation + mechanical ventilation	1 2 4	1.1 2.2 4.4

Conservative treatment and the use of hemostatic agents is rarely efficient in stopping the hemorrhage. Niu al. [40] have reported that pituitrin and thrombin may occasionally have good therapeutic effect in some cases of infantile bronchial Dieulafoy’s disease. Bronchoscopic ablation has been tried in a minority of cases [3] and is not without perils. Mediastinitis, esophageal injuries, and broncho esophageal fistulas are all potential complications [46]. Selective bronchial artery embolization is often performed as a first-line treatment and is efficient in most patients [64]. But hemoptysis may reoccur after the procedure. Among 56 patients treated with first-line SBAE, 24 underwent surgery afterwards due to unsuccessful embolization or to prevent reoccurrence of bleeding. In cases where intercostal artery embolization failed, authors reported that the abnormal blood vessels originated from the pulmonary artery, requiring pulmonary lobectomy to control the bleeding. Up to date, surgery has been the main definitive treatment with a success rate of nearly 100% in all reports [3]. Recurrence of hemoptysis in unlikely after resection of the diseased lung lobe.

## Conclusions

Dieulafoy’s disease of the bronchus is a rarely reported, and possibly underdiagnosed, cause of life-threatening hemoptysis. It should be included in the differential diagnosis of patients with massive hemoptysis with no other evident etiology. Bronchial angiography and EBUS may be highly suggestive of this disease. While SBAE is a less invasive procedure, surgical treatment remains a lifesaving approach that reduces the probability of recurrence. Therefore, it is the best choice which also allows an accurate histopathological diagnosis.

## Data Availability

Yes.

## List of abbreviations

BBD: bronchial Dieulafoy’s disease
CT: Computed tomography
EBUS: endobronchial ultrasound
SBAE: selective bronchial artery embolization

## References

[1] Dieulafoy G (1898). Exulceratio simplex. L’intervention chirurgicale dans les hématémèses foudroyantes consécutives à l’exulcération simple de l’estomac. Bull Acad Med.

[2] Sweerts M, Nicholson AG, Goldstraw P, Corrin B (1995). Dieulafoy’s disease of the bronchus. Thorax.

[3] Qian X, Du Q, Wei N, Wang M, Wang H, Tang Y (2019). Bronchial Dieulafoy’s disease: a retrospective analysis of 73 cases. BMC Pulm Med.

[4] van der Werf TS, Timmer A, Zijlstra JG (1999). Fatal haemorrhage from Dieulafoy’s disease of the bronchus. Thorax.

[5] Stoopen E, Baquera Heredia J, Cortes D, Green L (2001). Dieulafoy’s disease of the bronchus in association with a paravertebral neurilemoma. Chest.

[6] Maxeiner H (2001). Lethal hemoptysis caused by biopsy injury of an abnormal bronchial artery. Chest.

[7] Hope Gill B, Prathibha BV (2002). Bronchoscopic and angiographic findings in Dieulafoy’s disease of the bronchus. Hosp Med Lond Engl 1998.

[8] Bhatia P, Hendy M, Li Kam Wa E, Bowyer P (2003). Recurrent embolotherapy in Dieulafoy’s disease of the bronchus. Can Respir J.

[9] Kuzucu A, Gürses I, Soysal O, Kutlu R, Ozgel M (2005). Dieulafoy’s disease: a cause of massive hemoptysis that is probably underdiagnosed. Ann Thorac Surg.

[10] Pomplun S, Sheaff MT (2005). Dieulafoy’s disease of the bronchus: an uncommon entity. Histopathology.

[11] Löschhorn C, Nierhoff N, Mayer R, Zaunbauer W, Neuweiler J, Knoblauch A (2006). Dieulafoy’s disease of the lung: a potential disaster for the bronchoscopist. Respiration.

[12] Xie BS, Chen YS, Lin MF (2006). Dieulafoy’s disease of the bronchus: a case report and review of the literature. Zhonghua Jie He He Hu Xi Za Zhi.

[13] Rennert D, Gharagozloo F, Schwartz AM, Margolis M, Tempesta B, Wu J (2007). Dieulafoy’s lesion of the bronchus: report of a case and review of the literature. Pathol Case Rev.

[14] Fields EL, de Keratry DR (2008). Dieulafoy Disease of the Bronchus: Case Report and Presentation of a Novel Therapeutic Modality. J Bronchol Interv Pulmonol.

[15] Parrot A, Antoine M, Khalil A, Théodore J, Mangiapan G, Bazelly B (2008). Approach to diagnosis and pathological examination in bronchial dieulafoy disease: a case series. Respir Res.

[16] Zhu J, Chen G, Yin Y (2009). A case of bronchial Dieulafoy’s disease with massive hemoptysis. J Yunyang Med Coll.

[17] D’Souza F, Sharma R (2010). Dieulafoy’s disease of the bronchus. Pathol (Phila).

[18] Ding D, Lu L, Shuai Z (2010). A case of massive hemoptysis and asphyxia caused by Dieulafoy disease of bronchus biopsy and literature review. Int J Respir.

[19] Gurioli C, Casoni GL, Gurioli C, Tomassetti S, Romagnoli M, Ravaglia C et al. Endobronchial ultrasound in Dieulafoy’s disease of the bronchus: an additional application of EBUS. Monaldi Arch Chest Dis. 2010;73(4). 10.4081/monaldi.2010.287 21434565

[20] Hu H, Xin H, Xu E (2010). A case of massive hemoptysis caused by bronchial dieulafoy disease. Chin J Respir Crit Care Chin J Respir Crit Care.

[21] Wang W, Xia Y, Huang HD (2011). A case of Dieulafoy disease of bronchus and literature review. Int J Respir.

[22] Barisione EE, Ferretti GG, Ravera SS, Salio MM (2012). Dieulafoy’s disease of the bronchus: a possible mistake. Multidiscip Respir Med.

[23] Kolb T, Gilbert C, Fishman EK, Fishman E, Terry P, Pearse D (2012). Dieulafoy’s disease of the bronchus. Am J Respir Crit Care Med.

[24] Chen P, Fang N, Chen Z (2013). Bronchial mucosal biopsy leads to massive hemoptysis: a case report of bronchial artery abnormalities. Chin J Pract Intern Med.

[25] Yang RH, Li JF, Liu J (2013). Dieulafoy disease of the bronchus: 3 cases report with literature review. Zhonghua Jie He He Hu Xi Za Zhi.

[26] Fang Y, Wu Q, Wang B (2014). Dieulafoy’s disease of the bronchus: report of a case and review of the literature. J Cardiothorac Surg déc.

[27] Smith B, Hart D, Alam N (2014). Dieulafoy’s disease of the bronchus: a rare cause of massive hemoptysis. Respirol Case Rep juin.

[28] Liu Y, Li Y, Xing X (2014). Diagnosis and treatment of Dieulafoy’s disease of the bronchus. China J Endosc.

[29] Dalar L, Sökücü SN, Özdemir C, Büyükkale S, Altın S (2015). Endobronchial argon plasma coagulation for treatment of Dieulafoy Disease. Respir Care.

[30] Wang W, Chang XH (2015). A case of massive hemoptysis death caused by bronchial dieulafoy disease bronchoscopy biopsy. Clin Misdiagn Misther.

[31] Xia XD, Ye LP, Zhang WX, Wu CY, Yan SS, Weng HX (2015). Massive cryptogenic hemoptysis undergoing pulmonary resection: clinical and pathological characteristics and management. Int J Clin Exp Med.

[32] Ganganah O, Guo S, Chiniah M, Sah SK, Wu J (2015). Endobronchial ultrasound and bronchial artery embolization for Dieulafoy’s disease of the bronchus in a teenager: a case report. Respir Med Case Rep.

[33] Padilla-Serrano A, Estrella-Palomares V, Martínez-Palacios B, González-Spínola J (2015). A case of massive hemoptysis related to a smoking-history: an acquired form of the Dieulafoy’s disease?. Rev Port Pneumol.

[34] Wang Y, Zeng Y (2015). A case report of bronchial dieulafoy disease and literature review. Int J Respir.

[35] Zhang J, Ye J, Chen H (2015). A case of bronchial dieulafoy disease:diagnosed by airway ultrasound. Chin J Rural Med Pharmacy.

[36] Viola P, Villegas IA, Dusmet M (2016). Dieulafoy’s disease of the airways: a comprehensive review of a rare entity. Histopathology.

[37] Ge T, Wu H, Wang G (2016). Two cases of bronchial dieulafoy diseaseand literature review. Zhejiang Pract Me.

[38] Hadjiphilippou S, Shah PL, Rice A, Padley S, Hind M (2017). Bronchial dieulafoy lesion. A 20-Year history of unexplained hemoptysis. Am J Respir Crit Care Med févr.

[39] Madan K, Dhungana A, Hadda V, Mohan A, Guleria R (2017). Flexible bronchoscopic argon plasma coagulation for management of massive hemoptysis in bronchial Dieulafoy’s disease. Lung India Off Organ Indian Chest Soc.

[40] Niu HL, Yi P, Wang H, Wang FH, Liu W, Gao Q (2017). Infantile Dieulafoy’s disease of bronchus: report of a case. Zhonghua Bing Li Xue Za Zhi.

[41] Wadji MB, Farahzadi A (2017). Dieulafoy’s disease of the bronchial tree: a case report. Sao Paulo Med J.

[42] Yang D, Rong C, Gu J, Xu L, Zhang J, Zhang G (2017). Dieulafoy disease of the trachea with recurrent episodes of massive hemoptysis. Med (Baltim).

[43] Bonnefoy V, Garnier M, Tavolaro S, Antoine M, Assouad J, Fartoukh M, et al. Bronchial Dieulafoy’s Disease: visualization of embolization particles in bronchial aspirate. Am J Respir Crit Care Med. oct 2018;198(7):954–5. 10.1164/rccm.201711-2184IM 29877731

[44] Mincholé E, Penin RM, Rosell A (2018). The utility of Linear Endobronchial Ultrasound for the Incidental Finding of Dieulafoy Disease of the Bronchus. J Bronchol Interv Pulmonol.

[45] Pan F, Wang F, Liu Z (2018). The computed tomography angiography features of Dieulafoy disease of the bronchus. Zhonghua Jie He He Hu Xi Za Zhi.

[46] Sheth HS, Maldonado F, Lentz RJ (2018). Two cases of Dieulafoy lesions of the bronchus with novel comorbid associations and endobronchial ablative management. Med (Baltim).

[47] Wang F, Kuang TG, Wang JF, Yang YH (2018). A Rare cause of recurrent fatal hemoptysis: Dieulafoy’s disease of the bronchus. Chin Med J (Engl).

[48] Zhou F, Yan X, Liu R (2018). A case of misdiagnosis of bronchial dieulafoy disease. Clin Misdiagn Misther.

[49] Chen W, Chen P, Li X, Gao X, Li J (2019). Clinical characteristics and treatments for bronchial Dieulafoy’s disease. Respir Med Case Rep.

[50] Tang P, Wu T, Li C, Lv C, Huang J, Deng Z (2019). Dieulafoy disease of the bronchus involving bilateral arteries. Med (Baltim).

[51] White C, Ottaviano P, Munn N, Shweihat Y, Zeid F (2019). Massive hemoptysis due to recurrence of bronchial to pulmonary vascular malformation: a case report. Respir Med Case Rep.

[52] Zhou P, Yu W, Chen K, Li X, Xia Q (2019). A case report and review of literature of Dieulafoy’s disease of bronchus. Med (Baltim).

[53] Liao SX, Sun PP, Li BG (2020). A rare and fatal respiratory disease: bronchial Dieulafoy’s disease. Ther Adv Respir Dis.

[54] Yeh YT, Ramaswamy M, Shin J (2020). Bronchial Dieulafoy’s Disease in Children: a Case Report and Review of Literature. Front Pediatr.

[55] Giordano M, Bigazzi MC, Palladino MT, Russo MG (2020). A rare cause of massive hemoptysis in a child: Bronchial Dieulafoy’s disease - the first report of transcatheter treatment in pediatric age. Ann Thorac Med.

[56] Woodhull S, Bush A, Tang AL, Padley S (2020). Massive paediatric pulmonary haemorrhage in Dieulafoy’s disease: roles of CT angiography, embolisation and bronchoscopy. Paediatr Respir Rev.

[57] Li X, Chen J, Yang S (2020). Experience and literature review of 1 case of massive hemoptysis after bronchial Dieulafoy’s disease biopsy. J Rare Uncommon Dis.

[58] Vijayasekaran D, Sivabalan S (2021). Bronchial Dieulafoy Disease with recurrent life-threatening Hemoptysis. Indian Pediatr.

[59] Ruthberg JS, Abrol A, Howard NS (2021). Recurrent hemoptysis: a bronchial Dieulafoy’s lesion in a Pediatric patient. Ann Otol Rhinol Laryngol.

[60] Chen Y, Mao Y, Cheng X, Xiong R, Lan Y, Chen F (2021). Case Report: a case of Infant Bronchial Dieulafoy’s Disease and Article Review. Front Pediatr.

[61] Li J, Li L, He G (2021). A case of death from hemorrhagic shock caused by bronchial dieulafoy disease. Chin J Forensic Med.

[62] Tankeré P, Favrolt N, Yavordios S, Guerin AC, Georges M, Bonniaud P (2022). Hemorrhage in a patient with bronchial Dieulafoy’s disease and associated pulmonary fibrosis: a case report. Respir Med Case Rep.

[63] Locatelli T, Schneider T, Ottilinger T (2022). Intrabronchial haemorrhage: the bronchoscopist’s nightmare: a case of major haemoptysis due to pulmonary Dieulafoy’s disease. J Clin Images Med Case Rep.

[64] Xing X (2022). Research advances in Dieulafoy’s disease of the bronchus (review). Exp Ther Med.

[65] Walker CM, Rosado-de-Christenson ML, Martínez-Jiménez S, Kunin JR, Wible BC (2015). Bronchial arteries: anatomy, function, hypertrophy, and anomalies. Radiographics.

[66] Choi WS, Kim MU, Kim HC, Yoon CJ, Lee JH (2021). Variations of bronchial artery origin in 600 patients: systematic analysis with multidetector computed tomography and digital subtraction angiography. Med (Baltim).

